# The Role Played by Mitochondria in FcεRI-Dependent Mast Cell Activation

**DOI:** 10.3389/fimmu.2020.584210

**Published:** 2020-10-16

**Authors:** Maria A. Chelombitko, Boris V. Chernyak, Artem V. Fedorov, Roman A. Zinovkin, Ehud Razin, Lakhsmi Bhargavi Paruchuru

**Affiliations:** ^1^Belozersky Institute of Physico-Chemical Biology, Lomonosov Moscow State University, Moscow, Russia; ^2^Department of Cell Biology and Histology, Biology Faculty, Lomonosov Moscow State University, Moscow, Russia; ^3^Institute of Molecular Medicine, I.M. Sechenov First Moscow State Medical University, Moscow, Russia; ^4^Department of Biochemistry and Molecular Biology, School of Medicine, Hebrew University of Jerusalem, Jerusalem, Israel

**Keywords:** mast cell, mitochondria, FcεRI-dependent activation, IgE, allergy

## Abstract

Mast cells play a key role in the regulation of innate and adaptive immunity and are involved in pathogenesis of many inflammatory and allergic diseases. The most studied mechanism of mast cell activation is mediated by the interaction of antigens with immunoglobulin E (IgE) and a subsequent binding with the high-affinity receptor Fc epsilon RI (FcεRI). Increasing evidences indicated that mitochondria are actively involved in the FcεRI-dependent activation of this type of cells. Here, we discuss changes in energy metabolism and mitochondrial dynamics during IgE-antigen stimulation of mast cells. We reviewed the recent data with regards to the role played by mitochondrial membrane potential, mitochondrial calcium ions (Ca^2+^) influx and reactive oxygen species (ROS) in mast cell FcεRI-dependent activation. Additionally, in the present review we have discussed the crucial role played by the pyruvate dehydrogenase (PDH) complex, transcription factors signal transducer and activator of transcription 3 (STAT3) and microphthalmia-associated transcription factor (MITF) in the development and function of mast cells. These two transcription factors besides their nuclear localization were also found to translocate in to the mitochondria and functions as direct modulators of mitochondrial activity. Studying the role played by mast cell mitochondria following their activation is essential for expanding our basic knowledge about mast cell physiological functions and would help to design mitochondria-targeted anti-allergic and anti-inflammatory drugs.

## Introduction

Mitochondria are semi-autonomous double-membrane-bound organelles of an endosymbiotic origin with various compartments for operating the metabolic reactions including the citric acid cycle, oxidative phosphorylation (OXPHOS) and fatty acid β-oxidation. These reactions lead to the increase in the synthesis of ATP and also certain metabolites being generated. Furthermore, mitochondria produce ROS, accumulate Ca^2+^, and contribute to the programmed cell death and cell signaling regulation (1).

Increasing evidences suggests a strong correlation between cellular metabolism and mitochondrial morphology. For example, elongated mitochondria has contributed to a high level of OXPHOS activity and attenuated when the mitochondria was fragmented showing the impact of morphology (2, 3). It is well-known that when immune cells are activated their metabolism shifts from anabolism to catabolism and different immune cells employ different mechanisms of metabolic reprogramming enabling the optimized coordination between energetic and biosynthetic processes (3–6). Effector T cells and pro-inflammatory M1-macrophages predominantly employ anaerobic glycolysis which takes place in the cytoplasm, whereas regulatory T cell and anti-inflammatory M2-macrophages employ OXPHOS and fatty acid β-oxidation through mitochondria. This kind of alternative mechanism signifies the regulatory function of the mitochondrial morphology in these cells, where the mitochondria in effector T cells and M1-macrophages are fragmented while they were elongated in regulatory T cells and M2-macrophages (4, 5, 7). Moreover, immune cell activation is equally linked to the mitochondrial dynamics with in the cell. For example, antigen-induced activation of T cells is accompanied by fragmentation of mitochondria and their translocation to the immune synapse area (4, 5, 7). Mitochondrial fragmentation and translocation to the cell leading edge is necessary for migration of T cells (4, 8) and neutrophils (9). Efferocytosis of apoptotic cells by macrophages requires mitochondrial fragmentation as well (4).

The role played by mast cells in the development of inflammatory and allergic diseases is well-known (10, 11). Mast cell functions are mediated through a wide spectrum of biologically active compounds being secreted and regulated by various mechanisms. The most studied mechanism of mast cell activation is mediated by the interaction of antigens with immunoglobulin E (IgE) and a subsequent binding with the high-affinity receptor FcεRI. This event triggers FcεRI-dependent signaling which makes the mast cells secreting mediators such as histamine, proteoglycans, neutral proteases, and various cytokines. First, the preformed mediators are released from secretory granules via a degranulation process afterwards newly synthesized mediators such as cytokines and eicosanoids are being released to the extracellular environment for inducing inflammation (12, 13).

Mitochondria are actively involved in the FcεRI-dependent mast cell activation. Antigen-mediated mast cell stimulation is accompanied by mitochondrial fragmentation and their translocation to the site of exocytosis of secretory granules via secretion of mitochondrial components (14, 15). The OXPHOS process serves as an important step for mast cell degranulation and cytokine synthesis. The uncoupling of OXPHOS inhibits the FcεRI-dependent mast cell activation, however, the mechanisms that mediate this process is not fully understood (16–19). Mitochondria play an important role in regulating the cytosolic Ca^2+^ level which is critical for mast cell activation as they are able to accumulate Ca^2+^ (20–23). Mitochondrial ROS are also involved in activating mast cell (24, 25). Special attention should be given to the transcription factors STAT3 and MITF which play an important role in the development and function of mast cells. A small pool of these transcription factors reside in the mitochondria and modulate the mitochondrial activity independently as proteins other than regulating the expression of nuclear target genes (26–29).

Further investigation of the functions of mitochondria in mast cell activation is essential as it would expand our basic knowledge about mast cell physiology and could help in designing new anti-allergic and anti-inflammatory drugs.

## The FcεRI-dependent Mast Cell Activation

Mast cells represent an important cell population of the connective tissue that maintains its homeostasis and is involved in innate as well as adaptive immunity responses (12). The role played by these type of immune cells in the pathogenesis of various inflammatory and allergic diseases is well-documented (10). The strongest association of excessive mast cell activation and the severity of symptom manifestation were observed during arthritis, bronchial asthma, allergic rhinitis, and atopic dermatitis (11). Mast cell functions are mediated through the secretion of a wide spectrum of biologically active compounds. Mediators could be divided into two categories: the preformed mediators and the newly synthesized mediators. Mast cells secretory granules contain lysosomal proteins such as β-hexosaminidase mediators (such as histamine and serotonin), glycosaminoglycans (such as heparin and chondroitin sulfates) and enzymes (such as tryptase and chymase). The second group of mediators include metabolites of arachidonic acid, cytokines, chemokines, and growth factors are synthesized only upon mast cell stimulation (12, 13, 30, 31). There are two subpopulations of mast cells—mucosal mast cells, which are characterized by the presence of tryptase without chymase and mast cells of the connective tissue that contain both enzymes. Mucosal mast cells contain low levels of histamine, but produce many cysteinyl leukotrienes (LTC4, LTD4, LTE4, LTF4). Granules in these type of mast cells are characterized by their presence of chondroitin sulfate. In turn, connective tissue mast cells are characterized by higher content of histamine and secretes high levels of prostaglandin D2 while their granules contain heparin instead of chondroitin sulfate (32, 33). However, mast cells from different tissues are highly heterogeneous in the expression of both granules and enzymes needed for their secretion according to the studies on transcriptome analysis of bone marrow–derived mast cells (BMMCs) (34).

The mechanism of FcεRI-dependent mast cell activation is well-documented in number of reviews (12, 20, 35, 36). Interaction of the antigen-IgE complex with the FcεRI receptors induces their aggregation and phosphorylation of tyrosine residues in the (Ig) α immunoreceptor tyrosine–based activation motif (ITAM) regions of β- and γ-chains by the Lyn kinase. These regions bind to the spleen tyrosine kinase (Syk) which phosphorylates the transmembrane adaptor molecule linker for activation of T cells (LAT). LAT phosphorylation activates phospholipase Cγ (PLCγ) that catalyzes the hydrolysis of phosphatidylinositol (4,5)-bisphosphate (PIP_2_) in the plasma membrane. Inositol trisphosphate (IP_3_) and diacylglycerol (DAG) generated during hydrolysis of PIP_2_ will induce the release of Ca^2+^ from intracellular stores into the cytosol and activation of the protein kinase C (PKC) thus causing the degranulation of mast cell. Also, LAT activates the small GTPase RAS which in turn stimulates mitogen-activated protein kinase (MAPK) signaling leading to cytokine production and activates phospholipase A2 (PLA2) which regulates the synthesis of eicosanoids (12, 20, 35). Crosslinking of FcεRI receptors also activates Src-family kinase Fyn which is essential for mast cell degranulation along with cytokine and leukotriene production. Fyn kinase initiates complementary signals required for IgE-dependent mast cell degranulation and when there is a Fyn deficiency, an impaired FcεRI-dependent gene expression with a defective eicosanoid and cytokine production was resulted in mast cells. With the help of the adapter protein non-T cell activation linker (NTAL) Fyn phosphorylates Grb2-associeted binder 1 (Gab2) thus activating phosphatidylinositol 3 kinase (PI3K) (36, 37). Activated PI3K phosphorylates PIP_2_ and produces phosphatidylinositol (3,4,5)-trisphosphate (PIP_3_) which subsequently activates the 3-phosphoinositide-dependent protein kinase-1 (PDK1), RAC-alpha serine/threonine-protein kinase (Akt) and Bruton's tyrosine kinase (Btk) kinases (37, 38).

## The Role Played by Mitochondria in the FcεRI-Dependent Mast Cell Activation

### The Energy Metabolism

For quite a long time, mitochondria were thought to exert only the bioenergetic function by uptaking the substrate from cytosol and their catabolic conversion using fatty acid oxidation or the citric acid cycle (Krebs cycle) thus causing the reduction of nicotinamide adenine dinucleotide (NAD) and flavin adenine dinucleotide (FAD). Further oxidation of NAD hydrogen (NADH) and FAD dihydogen (FADH_2_) by the electron transport chain (ETC) is linked to a proton electrochemical potential generation across the inner mitochondrial membrane. The energy of this electrochemical potential is harnessed by the ATP-synthase to produce ATP. In addition to ATP production, mitochondria are involved in the biosynthesis of pyrimidines, certain fatty acids, heme, ROS production and maintenance of Ca^2+^ homeostasis (1).

Adequate ATP levels in mast cells have been shown to be essential for FcεRI-dependent activation of mast cells. ATP production and oxygen consumption was increased during antigen-induced activation (26). Apparently, both glycolysis and OXPHOS could be the source of ATP in mast cells (6). FcεRI-dependent stimulation of the rat basophilic leukemia RBL-2H3 mast cells was shown to induce phosphorylation and inactivation of the M2 pyruvate kinase (M2PK) which is involved in the terminal reaction of glycolysis and pyruvate production (39).

Using a Seahorse cell metabolism analyzer, the antigen-induced stimulation of mast cells derived from the murine bone marrow has been shown to rapidly stimulate the glycolysis (40). Glycolysis suppression by dichloroacetate that inhibits the kinase of pyruvate dehydrogenase and ETC complex I inhibition by rotenone abated both degranulation and cytokine production. The competitive inhibitor of glycolysis 2-deoxyglucose also diminished both degranulation and production of cytokines upon antigen-dependent mast cell activation. However, the suppressing fatty acid oxidation by etomoxir which inhibits carnitine palmitoyltransferase-1 had no effects (40). It should be noted that mast cell sensitization only with IgE prior to antigen-induced stimulation elevates the glycolytic capacity (40). Our own data demonstrated that inhibition of glycolysis doesn't affect FcεRI-dependent degranulation of RBL-2H3 and bone marrow-derived mast cells. This indicated that the major part of the energy for degranulation is derived from mitochondrial ATP (26).

The PDH complex serves as a gatekeeper in the metabolism of pyruvate in order to maintain glucose homeostasis during the fed and fasting states. Important mast cell functions such as degranulation and cytokine secretion were found to be regulated by mitochondrial PDH. This is based on our findings that in IgE-antigen activated RBL-2H3 cells and BMMCS, serine 293 dephosphorylated PDH levels were significantly reduced, indicating the active state of this complex. Furthermore, CPI-613, a small molecule selective inhibitor of PDH, which is now in phase 2 of clinical studies for cancer treatment has severely impaired mitochondrial ATP production. This inhibitor treatment led to a 50% decrease in mast cell degranulation and completely abolished the tumor necrosis factor alpha (TNFα) secretion in RBL-2H3 cells. This effect was not a result of apoptosis as no cleaved caspase 3 was observed and was due to PDH inactivation where a significant increase in PDH phosphorylation levels indicates a decrease in the complex's activity. Moreover, the knockdown of PDH using small interfering ribonucleic acid (RNA) (siRNA) has also replicated the degranulation results. PDH depletion has greatly effected interleukin 6 (IL-6) and TNF-α levels than degranulation values which indicates a strong correlation between the loss of mitochondrial ATP and cytokine secretion during mast cell exocytosis. These findings were extended to human cord blood-derived mast cells also where both degranulation and IL-6 secretion were abolished by CPI-613, providing a future potential target for allergic diseases research (28).

The antigen-dependent stimulation of mast cell was shown to effect the extracellular signal-regulated kinase (Erk1/2) dependent phosphorylation of mitochondrial STAT3 thus causing an increase in OXPHOS activity. Inhibition of STAT3 activity has attenuated both degranulation and cytokine secretion in mast cells (26, 29). Thus, FcεRI-dependent mast cell stimulation requires both activities of glycolysis and OXPHOS.

### The Mitochondrial Membrane Potential

The mitochondrial membrane potential (δΨm) plays an important role in the regulation of FcεRI-mediated mast cell degranulation. ΔΨm is generated by ETC proton pumps (complexes I, III, and IV). Along with the proton gradient, ΔΨm forms the proton electrochemical transmembrane potential (or the proton-motive force) used for ATP synthesis (41, 42). ΔΨm is a driving force for the transporting various substrates in mitochondria and for Ca^2+^ accumulation. High ΔΨm can induce NAD reduction (so-called reverse electron transport) that is the key contributor to mitochondrial ROS production (41–43). Membrane potential plays an important role in signal transduction and regulates the mitochondrial structural dynamics. Decreased ΔΨm is accompanied by fragmentation of the elongated mitochondria (41, 42). A significant decline of mitochondrial potential is associated with mitochondrial dysfunction. It activates the mitochondrial quality control systems including the phosphatase and tensin homolog induced kinase 1(PINK1)-parkin pathway which induces selective autophagy of defective mitochondria (mitophagy). At high ΔΨm PINK1 undergoes degradation, while ΔΨm is reduced, PINK1 accumulates on the outer surface of the mitochondria and recruits parkin ubiquitin ligase that ubiquitinylates PINK1 leading to mitophagy induction (41–43). ΔΨm decrease due to OXPHOS uncoupling attenuates mast cell activation. The uncoupler carbonyl cyanide m-chlorophenyl hydrazone (CCCP) inhibits the antigen-induced secretion of β-hexosaminidase in rat mast cell line RBL-2H3 (16). Mitochondrial respiration inhibitors such as ETC complex I inhibitor rotenone, complex III inhibitor antimycin A, and the uncoupler carbonyl cyanide-4-(trifluoromethoxy)phenylhydrazone (FCCP) suppress IgE-mediated degranulation of murine bone marrow mast cells and RBL-2H3 cells. This suggests that this process may depend on a high ATP level. However, antimycin A and FCCP (but not rotenone) promoted degranulation in the absence of extracellular Ca^2+^ via a rapid decrease of ΔΨm. Mitochondrial depolarization enhanced IgE-mediated Ca^2+^ release from mitochondria and intracellular stores. This can imply that uncouplers affect mast cell degranulation both via a decreased ATP level and an increased release of mitochondrial Ca^2+^ (17).

Many natural and synthetic compounds possess an uncoupling activity along with the uncouplers widely used in experimental work. The antibacterial and antifungal agent triclosan has an uncoupling activity. Triclosan brings about a decline in ΔΨm and ATP production and promotes mitochondrial fragmentation and ROS generation in unstimulated RBL-2H3 cells. Upon mast cell stimulation, triclosan inhibits microtubule polymerization and mitochondrial translocation to the plasma membrane and suppresses the entry of extracellular Ca^2+^ through the plasma membrane. The inhibited mitochondrial translocation might prevent the activation of calcium release-activated channels (CRAC) which plays a crucial role in Ca^2+^ from the extracellular environment indicates thattriclosan attenuates FcεRI-dependent mast cell degranulation (18, 19).

It is worth noting that some uncouplers can act as mast cell activators. In our recent study, we have shown that degranulation of RBL-2H3 mast cells is stimulated by usnic acid which is a secondary messenger in lichens and has an uncoupling activity. Usnic acid appears to act not only as a protonophore uncoupler but also as a calcium ionophore. Similar to calcium ionophore A23187, usnic acid elevated the intracellular Ca^2+^ level in RBL-2H3 cells acting as a trigger for degranulation (44). Thus, ΔΨm plays an important role in mast cell function, most likely due to its role in Ca^2+^ transport across the mitochondrial membrane.

### Calcium Signaling

All stages of FcεRI-dependent mast cell activation including their degranulation, biosynthesis of eicosanoids and cytokine production are Ca^2+^-dependent (20–23, 45). Antigen-induced mast cell stimulation activates phosphoinositide phospholipase C (PLCγ) which catalyzes phosphatidylinositol 4,5-bisphosphate (PIP_2_) hydrolysis in the plasma membrane. Inositol trisphosphate (IP_3_) induces Ca^2+^ release from the endoplasmic reticulum through inositol trisphosphate receptor (IP_3_R) channels into the cytosol (20–23, 45). Mitochondria and endoplasmic reticulum can strongly interact with each other via special contacts called mitochondria-associated membranes (MAMs) (22) with their membranes that are spaced 10–50 nm apart. Ca^2+^ released from endoplasmic reticulum penetrates into mitochondria through the potential-dependent porin voltage-dependent anion channels (VDAC) localized at the outer mitochondrial membrane following mitochondrial calcium uniporter (MCU) at the inner mitochondrial membrane (22, 45). ATP-dependent Ca^2+^ pumps sarco/endoplasmic reticulum Ca2+-ATPase (SERCA) restore the Ca^2+^ pool in endoplasmic reticulum (22). The downregulation of the MCU transporter has been shown to impair FcεRI-dependent mast cell degranulation (21). An increased level of mitochondrial Ca^2+^ enhances OXPHOS and promotes ATP production and ROS generation. Importantly, Ca^2+^ release from mitochondria, mediated by Na^+^/Ca^2+^-exchanger and to a lesser extent by H^+^/Ca^2+^-exchanger, is important for maintaining of Ca^2+^ oscillations in the cytosol and preventing the mitochondrial Ca^2+^ overload (20–23, 45). Excessive accumulation of Ca^2+^ in mitochondria promotes the opening of the non-selective mitochondrial permeability transition pore (mPTP) which is essential for a rapid Ca^2+^ release from mitochondria. The mPTP complex most likely comprises ATP-synthase (F0F1), adenine nucleotide translocase (ANT) and cyclophilin D from the mitochondrial matrix (46). Both decreased ΔΨm and oxidative stress significantly increases the probability of mPTP opening which plays an important role in mast cell activation. This is supported by the fact that atractyloside and bongkrekic acid (the agonist and antagonist of mPTP, respectively) enhanced and suppressed FcεRI-mediated intracellular Ca^2+^ release in mast cells, respectively. Bongkrekic acid abolished the elevated antigen-induced mast cell degranulation which was observed in the absence of intracellular Ca^2+^ upon treatment with antimycin A or FCCP (17).

It is known that mPTP opening can be reversible and facilitate the long-term Ca^2+^ oscillations in the cytoplasm. Meanwhile, prolonged mPTP opening results in reduced ATP production and increased ROS generation, mitochondrial swelling, disruption of the outer mitochondrial membrane, and release of proapoptotic factors from the mitochondrial intermembrane space into the cytoplasm (47).

Transient Ca^2+^ oscillations triggered by Ca^2+^ release from the intracellular stores are incapable of maintaining the degranulation of antigen-stimulated mast cells in the absence of the extracellular Ca^2+^ influx. Mast cell activation requires Ca^2+^ influx by store-operated entry (SOCE). SOCE is activated by Ca^2+^ depletion in the endoplasmic reticulum and subsequent influx of extracellular Ca^2+^. The loss of Ca^2+^ binding to EF-hand motifs in a luminal domain of Ca^2+^ sensor stromal interaction molecule 1 (STIM1) results in its oligomerization and redistribution to regions of the endoplasmic reticulum proximal to the plasma membrane where it interacts with pore-forming subunits of calcium release-activated channel (Orai1/CRACM1) forming an active CRAC (20, 48). Besides CRAC some transient receptor potential canonical (TRPC) TRPCs can participate in Ca^2+^ influx in FcεRI-dependent mast cell activation. For example, mast cell Fyn kinase regulates expression of TRPC1. Fyn null mast cells demonstrate a decreased expression of TRPC1, impaired Ca^2+^ influx and cortical F-actin depolymerization (a key step for granule-plasma membrane fusion) has resulted in a decreased degranulation (49). TRPC5 in RBL-2H3 cells associates with STIM1 and Orai1, enhances entry of Ca^2+^ and degranulation (50). The results obtained on the studies of non-mast cells suggest that interaction between STIM1 and Orai1 can be mediated by microtubule-directed reorganization of endoplasmic reticulum and establishment of contacts between the endoplasmic reticulum and the plasma membrane (51–53).

Mitochondria appears to play an important role in SOCE. Trafficking of STIM1 to endoplasmic reticulum-plasma membrane junctions and subsequent activation of CRAC in rat basophilic leukemia cells RBL-1 and HEK293 cells was impaired following the mitochondrial depolarization (54). The mitochondrial Ca^2+^ current was shown to be necessary for STIM1 oligomerization and activation in HeLa cells (55). Two steps elevation in mitochondrial Ca^2+^ concentration was shown in antigen-activated RBL-2H3 mast cells. The first is Ca^2+^ entry to mitochondria derived from the Ca^2+^ release from the endoplasmic reticulum. The second step is Ca^2+^ influx mediated by STIM1-Orai1. Inhibition of mitochondrial ETC complex I and III by rotenone and antimycin A, respectively, diminished mitochondrial Ca^2+^ uptake and inhibited antigen-induced degranulation (56). Mitochondrial Ca^2+^ uptake can maintain an active state of CRAC channels along with activation of OXPHOS and STIM1. Studies on T-lymphocytes have demonstrated that increased Ca^2+^ levels will lead to CRAC inactivation and the mitochondrial Ca^2+^ uptake in the vicinity of CRAC prevents their inactivation (8). Another mechanism that maintains the extracellular Ca^2+^ influx was demonstrated on HEK293T and RBL-2H3 cells, where the activity of mitochondrial Na^+^/Ca^2+^-exchanger has interfered the ROS-dependent Orai1 inactivation (57).

When intracellular Ca^2+^ in mast cells is elevated, it activates Ca^2+^-binding protein calmodulin which in turn stimulates calmodulin-dependent protein kinase (CaMK), myosin light chain kinase (MLCK), and phosphatase calcineurin (47). The importance of CaMK and MLCK activation for secretory granule exocytosis was demonstrated in the RBL-2H3 mast cells (58). Calcineurin promotes mitochondrial fragmentation and induces nuclear translocation of the transcription factors such as transcription factor EB (TFEB), nuclear factors of activated T cell (NFAT) and nuclear factor- kappaB (NF-kB) which are involved in autophagy and mitochondrial biogenesis as well as secretion of pro-inflammatory cytokines (59, 60). The activation of transcription factor NFAT, which regulates the expression of pro-inflammatory cytokines associated with the Th2-dependent immune response, strongly depends on calcineurin. The inhibited calcineurin activity reduces the secretion of both preformed and synthesized mediators. It should be noted that calcineurin inhibitors are used for therapy of atopic dermatitis and some other allergic diseases. NFAT has been shown to regulate mast cell degranulation affecting the activity of kinases protein kinase C (PKC), p38, and Erk (59, 60).

The soluble NSF attachment proteins receptor (SNARE) protein complex plays a crucial role in fusing the secretory granules with each other and with the plasma membrane. Some proteins required for its proper function are Ca^2+^-sensitive. Such proteins include Munc13-4, synaptotagmin II, and double C2 (Doc2α) (61–63).

### Mitochondrial ROS

We have already discussed the role played by ROS in mast cell activation (25) and the present review focuses on the possible role of the mitochondrial ROS in mast cell activation.

Most mitochondrial ROS arise from ETC function (by complexes I and III, NADH dehydrogenase and cytochrome bc1 complex, respectively). Mitochondrial ROS generation depends on many factors: oxygen level, respiration rates, Ca^2+^ levels, and ΔΨm. For example, high ΔΨm may boost ROS production. Mitochondrial ROS can be generated as byproducts of dehydrogenase activity in the mitochondrial matrix, the proteins having Src homology 2 (p66shc) in the intermembrane space, monoamine oxidase at the outer mitochondrial membrane, and NADH oxidase (NOX4) at the inner mitochondrial membrane. Mitochondria have an efficient antioxidant system. It includes thiol-containing peptide glutathione, thioredoxins and glutaredoxins, glutathione reductases, thioredoxin peroxidases (peroxiredoxins), and superoxide reductases (64–66).

Histamine production and mast cell degranulation were shown to be attenuated by uncoupling protein 2 (UCP2), the protein of the inner mitochondrial membrane that regulates ROS generation (24). Our previous data indicated that mitochondria-targeted antioxidant SkQ1 abrogates the antigen-dependent degranulation of RBL-2H3 mast cells (67), which implies that mitochondrial ROS contribute to mast cell activation. As it will be discussed below in the part “Mitochondrial dynamics,” mast cell degranulation is accompanied by the mitochondrial fragmentation and its translocation to the plasma membrane. Preventing mitochondrial fragmentation suppresses mast cell degranulation (14). Mitochondrial ROS seem to be a vital regulator of mitochondrial fragmentation. The mitochondria-targeted antioxidants MitoQ (68–70) and SkQ1 (71) can inhibit mitochondrial fragmentation induced by uncoupling agents and oxidative stress in many cell cultures. The protective effect of the mitochondria-targeted antioxidants appears to be mediated by modulation of the activity or localization of dynamin-like proteins Drp1 and OPA1 (66, 69), as well as by regulation of the expression of genes that control mitochondrial dynamics (70, 72).

Probably, mitochondrial ROS can regulate exocytosis of mast cells by acting on Ca^2+^ channels in secretory granules. The main lysosomal Ca^2+^ channel mucolipin-1 (MCOLN1/TRPML1) is localized in the secretory lysosomes of NK cells related to secretory granules (73) and can be directly activated by mitochondrial ROS (61, 74, 75). Ca^2+^ release via TRPML1 leads to lysosomal fission (76) and calcineurin-dependent translocation of the nuclear transcription factor TFEB which stimulates lysosome exocytosis, autophagy, and lysosome biogenesis (61, 74, 75). The key role of mucolipin-1 in the regulation of granule exocytosis was demonstrated in experiments in which the pharmacological inhibition of phosphoinositide kinase PIKfyve, which stimulates the opening of the mucolipin-1 channel, inhibited exocytosis in mouse bone marrow-derived mast cells during FcεRI-dependent activation (77).

The results obtained from the studies of various cell types suggest that mitochondrial ROS can also affect antigen-dependent mast cell activation by modifying activities of MAPK and transcription factors NF-κB and NFAT. Stimulating the mitochondrial ROS production by antimycin A induces Erk1/2 phosphorylation and interleukin 8 (IL-8) secretion by polymorphonuclear leukocytes from human peripheral blood (78). There is evidence indicating that mitochondrial ROS are involved in activating P38 MAPK (79, 80) and c-Jun N-terminal kinases (JNK) (81). Mitochondria-targeted antioxidants can inhibit the phosphorylation of all three mentioned kinases (81–83).

Mitochondrial ROS can stimulate NF-κB signaling by activating the kinase (IKK) of the inhibitor of NF-κB (IκB), which promotes its proteasome degradation and induces nuclear translocation of NF-κB (81, 84). Mitochondrial ROS-dependent activation of IKK can be mediated by several mechanisms, including the formation of intermolecular disulfide bonds in NF-κB essential modulator (NEMO), a component of the IKK complex (85).

The activity of another important transcription factor, NFAT, also depends on mitochondrial ROS. It has been shown that NFAT nuclear translocation is abolished in T cells deficient in the mitochondrial ubiquinol-cytochrome C reductase subunit, the Riske iron-sulfur protein (Uqcrfs1), which is involved in the generation of ROS (86).

**Figure 1:**
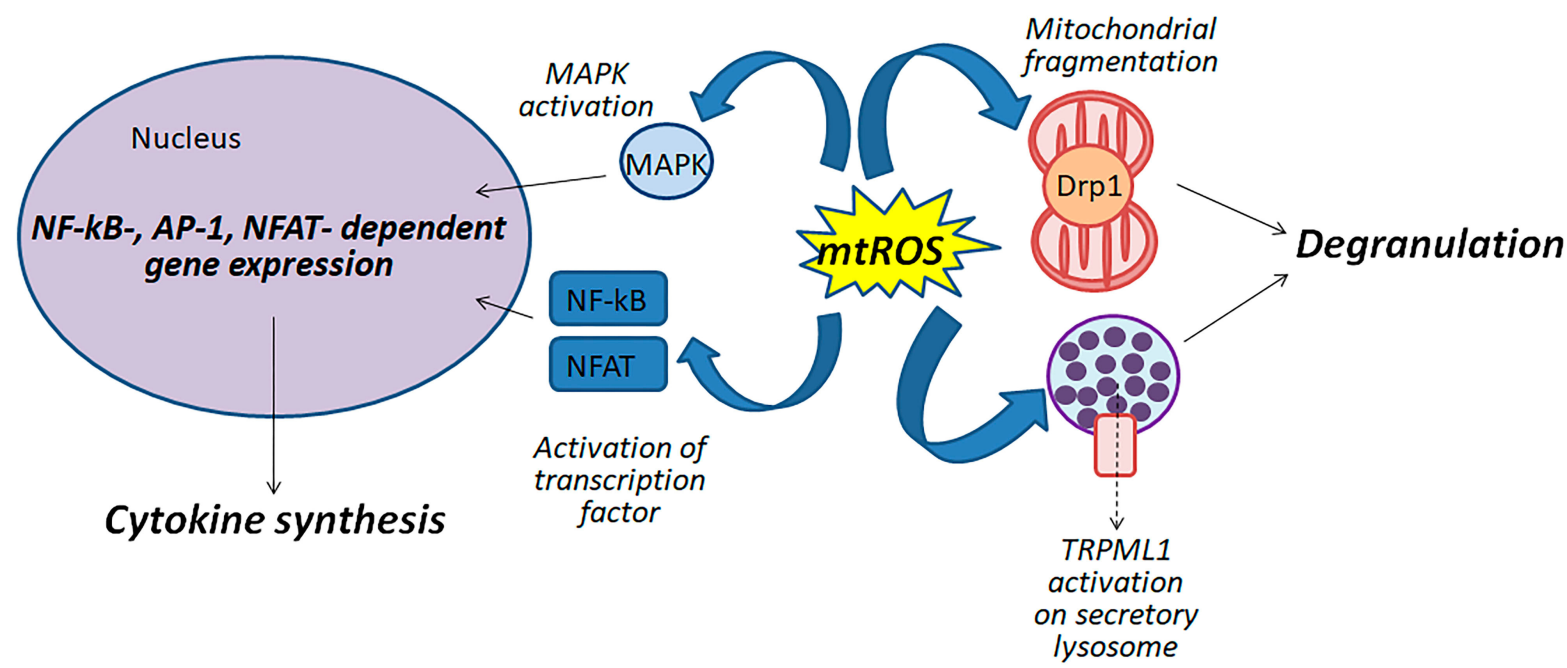
Mitochondrial ROS (mtROS) can be involved in the activation of mast cells by various mechanisms: (i) participating in the fragmentation of mitochondria; (ii) regulating degranulation by stimulation of Ca^2+^-channels TRPML1 localized to secretory lysosomes; (iii) affecting the activity of MAP kinases and transcription factors NF-κB and NFAT.

Figure 1 shows schematically possible function of mitochondrial ROS in the antigen-dependent mast cell stimulation.

### The Mitochondrial Dynamics

The research evidences indicates that mitochondrial reticulum is a highly dynamic structure. Depending on the cell requirements, mitochondria can both alter their localization using the cytoskeleton and modulates their morphology by fusion and fragmentation. Mitochondrial fusion is regulated by the GTPases mitofusin1 (Mfn1) and mitofusin2 (Mfn2) at the outer mitochondrial membrane and by OPA1 at the inner mitochondrial membrane. Mitochondrial fragmentation is mediated mainly by the GTPase Drp1 which is localized in the cytosol is being inactive (2, 3).

Inhibiting Drp1 activity by mitochondrial division inhibitor 1 (mDivi-1) or downregulation of Drp1 expression using siRNA suppresses mitochondrial fragmentation and translocation, attenuates mast cell degranulation but does not affect *de novo* biosynthesis of mediators. The translocation of mitochondria to the plasma membrane was observed in the mast cells isolated from the skin of atopic dermatitis patients. Furthermore, Drp1 and calcineurin were upregulated in the skin of those patients (14). The number of cristae in mitochondria of RBL-2H3 mast cells was found to increase upon the antigen-induced stimulation (87). Generally, the number of cristae increases as OXPHOS activity enhances (7). Furthermore, IgE-dependent stimulation of laboratory of allergic diseases 2 (LAD2) mast cells was shown to be accompanied by secretion of mitochondrial particles, mitochondrial DNA, and ATP in the absence of cell death (15).

Drp1 activity depends on its phosphorylation on Ser616 and Ser637. Dephosphorylation of Drp1 on Ser637 by Ca^2+^-dependent phosphatase calcineurin promotes the recruitment of Drp1 to the mitochondrial surface. Drp1 activation requires phosphorylation on Ser616 which is executed by various MAPK kinases including Erk1/2 (2). Calcineurin activation occurs in response to an increased intracellular Ca^2+^. Apart from Drp1 activation, calcineurin triggers nuclear translocation of transcription factors TFEB, NFAT, and NF-kB involved in the biogenesis of mitochondria and lysosomes, autophagy, and secretion of pro-inflammatory cytokines. Activation of the NFAT transcription factor that regulates the expression of the pro-inflammatory cytokines involved in T helper type 2 (Th2) dependent immune response strongly depends on calcineurin (60).

The role of the Drp1-dependent reorganization of mitochondrial reticulum in T cell activation is well-studied. Mitochondrial fragmentation that occurs during the differentiation of effector T cells is accompanied by a disassembly of ETC complexes and reducing OXPHOS activity (7). Upon T cell activation, mitochondria are translocated to the area of the immunological synapse. Mitochondrial Ca^2+^ uptake interferes with Ca^2+^-dependent inactivation of CRAC channels and thus facilitates the increased and stabilized extracellular Ca^2+^ influx (88). The Erk1/2-dependent phosphorylation of Drp1 and the subsequent mitochondrial fragmentation were shown to be necessary for the T cell migration as it requires local ATP production at the cell leading edge and activation of the motor protein myosin (89, 90).

Notably, that Drp1 can exert functions distinct from the regulation of mitochondrial fragmentation. Drp1 has been shown to be involved in postsynaptic endocytosis in neurons (91). Drp1 was also shown to be involved in the pore formation for exocytosis of thrombocyte granules (92).

These data suggest that Drp1-mediated mitochondrial fragmentation upon antigen-induced mast cell stimulation can regulate degranulation by maintaining Ca^2+^ homeostasis and the local ATP production. At the same time, the effects of Drp1 on mast cell degranulation may be associated not only with the influence of Drp1 on mitochondrial fragmentation but also with its direct role in the exocytosis.

### The Mitochondrial STAT3 and MITF

It's important to discuss the issue of two transcription factors, STAT3 and MITF, which have a small pool localized in mast cell mitochondria. The transcriptional switch induced by these proteins allows the cells to shift their metabolism rapidly in response to altered conditions.

FcεRI-dependent mast cell activation is accompanied by Erk1/2-dependent phosphorylation of STAT3 on Ser727 and its translocation to mitochondria. This affects the ETC complex III activity and elevates ATP production, but the influence on the activity of complexes I and II cannot be ruled out. The selective small-molecule inhibitor of STAT3 Stattic and the mitochondria-targeted inhibitors Mitocur-1 and Mitocur-3 (the curcumin conjugated with the triphenylphosphonium lipophilic cations) suppress both degranulation and cytokine production by mast cells *in vitro* and *in vivo* (26, 29).

Mitochondrial STAT3 can modulate mast cell activation affecting ETC activity, ROS production, Ca^2+^ homeostasis, and mitophagy. Rotenone-induced mitochondrial ROS mediate phosphorylation of STAT3 on Ser727 and its subsequent translocation to mitochondria. In its turn, mitochondrial STAT3 facilitates ATP production affecting predominantly the activity of ETC complexes I and II and decreases ROS generation. Therefore, STAT3 senses and regulates ROS levels (27, 93). Mitochondrial STAT has been also shown to bind to the mPTP component cyclophilin D and prevent its opening which can be one of the crucial mechanisms of ROS generation inhibition (94). Furthermore, mitochondrial STAT3 was shown to increase the mitochondrial and intracellular levels of Ca^2+^ (27). There is also evidence indicating that mitochondrial STAT3 inhibits mitophagy (95).

Pyruvate dehydrogenase complex (PDC) is the main regulator of Kreb's cycle and it's a complex made up of 3 subunits including pyruvate dehydrogenase PDH (2 subunits E1α, β), dihydrolipoamide acetyltransferase (E2) and dihydrolipoamide dehydrogenase (E3). For the conversion of pyruvate to acetyl-CoA, an activated dephosphorylated PDC catalyzes the oxidative decarboxylation step, thus regulating the citric acid cycle inside mitochondria. For the mast cell function during FcεRI-dependent activation an increase in OXPHOS activity and ATP production was observed (26). MITF whose major function is to regulate the differentiation of melanocytes and osteoclasts (96) was also found in mast cells (97). The studies on lysyl-tRNA synthetase (LysRS), diadenosine tetraphosphate (Ap4A), histidine triad nucleotide-binding protein 1 (HINT1) in FcεRI-dependent stimulated mast cells highlights the transcriptional activity of MITF in mast cells and describes the LysRS-Ap4A-HINT-MITF signaling pathway (98, 99). Interestingly, peroxisome proliferator–activated receptor γ coactivator 1 α (PGC-1 α) an important coactivator for regulating OXPHOS genes for maintaining the mitochondrial biogenesis was found to be regulated by MITF (100) in melanoma cells. Most recently MITF as a protein was found to be partially localized in the mitochondria and found to interact with pyruvate dehydrogenase E1α subunit (PDH E1α) in mast cells. Allergic stimulation has activated the PDH thus causing its detachment from MITF in IgE-DNP treated RBL-2H3 cells and bone marrow–derived mast cell. Also an impaired degranulation, ATP production, oxygen consumption and the reduction of TNFα and IL-6 cytokines with CPI-613 (PDC inhibitor) treatment in cord blood–derived mast cell explains the importance of PDH complex during mast cells function. A decreased PDH activity with MITF overexpression in bone marrow–derived mast cell and RBL-2H3 cells indicates the importance of protein interactions in mitochondria. Also, MITF was found to associate with phosphorylated PDH after CPI-613 treatment clearly emphasizes the regulatory role of MITF for maintaining the PDH activity during allergic stimulus (28). However, the mitochondrial MITF localization and function other than regulating the PDC activity during allergic activation remains unclear.

## Conclusion and Future Directions

**Figure 2:**
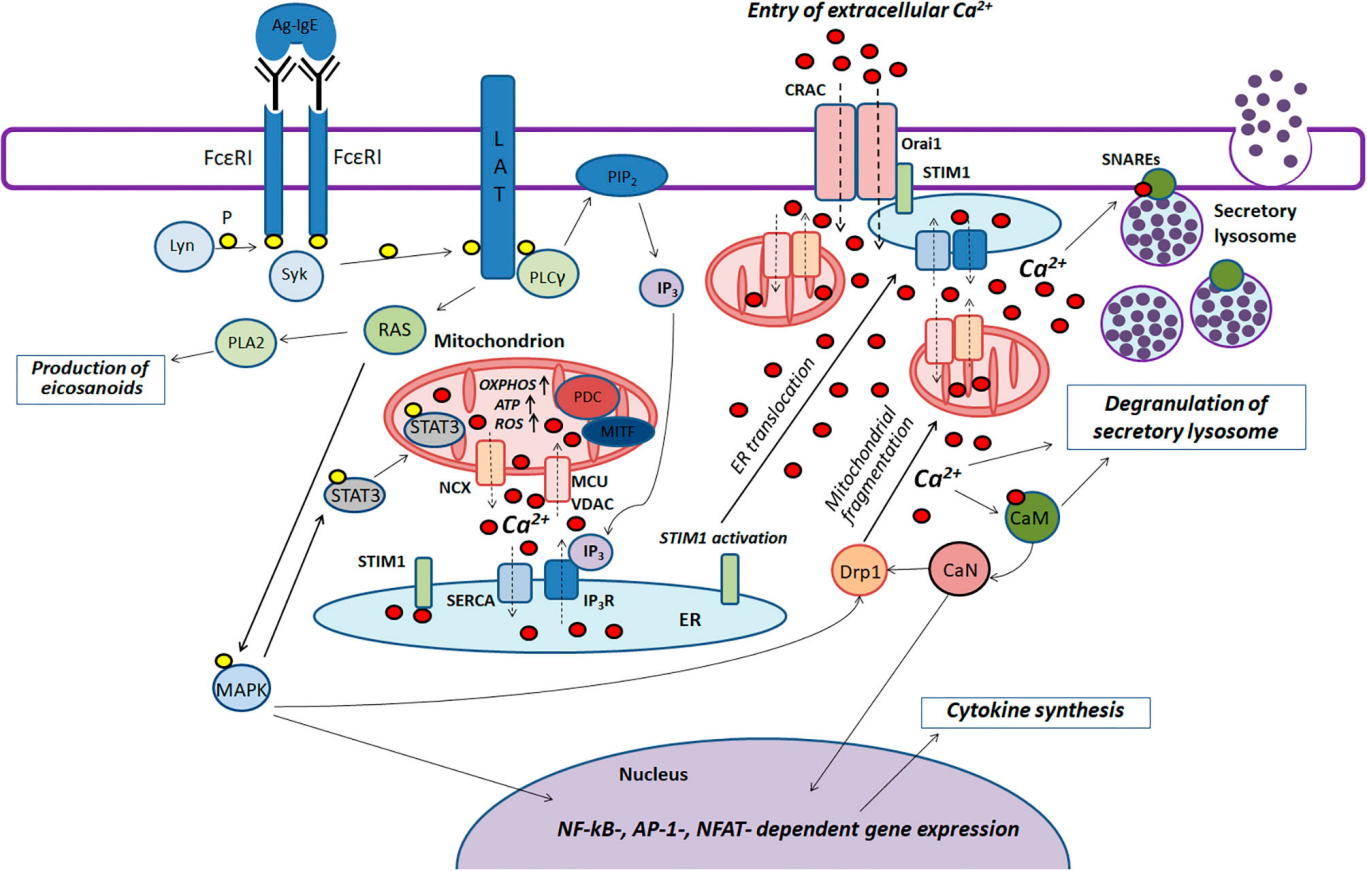
The role played by mitochondria in the antigen-dependent mast cell stimulation. Binding of the antigen complex to IgE (Ag-IgE) causes aggregation of FcεRI receptors and recruits the Lyn kinase which phosphorylates (P) ITAM regions of FcεRI with subsequent recruitment of the Syk kinase. This kinase activates the adapter molecule LAT that further stimulates phospholipase PLCγ and small GTPase RAS. PLCγ hydrolyzes PIP_2_ with the production of IP_3_ and DAG. IP_3_ causes Ca^2+^ release from endoplasmic reticulum (ER) through IP_3_R channels. Mitochondria interact with ER and uptake the released Ca^2+^ via the potential-dependent transporter system VDAC-MCU. In mitochondria, Ca^2+^ enhances OXPHOS and ROS and ATP production. Local Ca^2+^ oscillations occur due to the activity of the mitochondrial Na^+^/Ca^2+^-exchanger (NCX) and SERCA pumps localized in ER. Ca^2+^ release from ER causes activation and oligomerization of the calcium sensor STIM1. After the microtubule-dependent ER translocation to the plasma membrane, STIM1 binds to the pore-forming subunits of CRAC Orai1. This maintains the current of the intracellular Ca^2+^. The activated GTPase RAS stimulates phospholipase PLA2 and activates MAPK signaling cascade leading to the biosynthesis of eicosanoids and cytokine production. Furthermore, MAPK kinase Erk1/2 induces phosphorylation of the transcription factor STAT3 and its translocation to mitochondria. There it enhances OXPHOS and ATP production but prevents excessive ROS generation and mitophagy. A pyruvate dehydrogenase complex (PDC) regulating mitochondrial MITF along with STAT3 could also play a potential role in regulating the antigen-induced stimulation of mast cells. An elevated level of intracellular Ca^2+^ activates calmodulin (CaM) followed by stimulation of the phosphatase calcineurin (CaN) which together with MAPK kinases induces Drp1-dependent mitochondrial fragmentation along. Fragmented mitochondria are translocated to the plasma membrane. Suppression of fragmentation or translocation of mitochondria attenuates the antigen-dependent mast cell degranulation. The main function of mitochondria seems to be as following: they maintain intracellular Ca^2+^ current preventing of Ca^2+^- and ROS-dependent inactivation of the CRAC channels. The accumulation of the intracellular Ca^2+^ is also important for activating certain proteins that play an important role in SNARE complex function which is necessary for fusion of the secretory lysosomes with each other and with the plasma membrane.

Mast cells play an important role in the pathogenesis of various inflammatory and allergic diseases. Mitochondria are actively involved in many stages of FcεRI-dependent mast cell activation. Based on literature data summarized in this review we try to present scheme of the function of mitochondria in the antigen-dependent mast cell stimulation (Figure 2).

Investigation of the functions of mitochondria in mast cell activation is essential as it would expand the basic knowledge about mast cell physiology and would help to design new anti-allergic and anti-inflammatory drugs targeted to mitochondria. The data summarized in the review indicate the promising potential of uncouplers, mitochondria-targeted antioxidants, and Drp1 and STAT3 inhibitors to reduce FcεRI-activation of mast cells. However, a wealth of information was obtained solely from *in vitro* studies of mast cell degranulation without estimation of cytokines and eicosanoids secretion. There is an urgent need for *in vivo* research to investigate the therapeutic potential of the drugs targeted to mitochondria.

## Author Contributions

All authors contributed to manuscript designing, writing, and editing and reviewed the final version of this article.

## Conflict of Interest

The authors declare that the research was conducted in the absence of any commercial or financial relationships that could be construed as a potential conflict of interest.

## Abbreviations

ANT: adenine nucleotide translocase
Ap4A: diadenosine tetraphosphate
ATP: adenosine triphosphate
BMMCs: bone marrow–derived mast cells
Ca^2+^: calcium ions
CaMK: calmodulin-dependent protein kinase
CRAC: calcium release-activated channels
DAG: diacylglycerol
DNP: 2,4-dinitrophenol
Doc2α: double C2
Drp1: dynamin related protein 1
Erk1/2: extracellular signal-regulated kinase
ITAM: (Ig) α immunoreceptor tyrosine–based activation
ETC: electron transport chain
FAD: flavin adenine dinucleotide
FADH_2_: FAD dihydogen
FCCP: carbonyl cyanide-4-(trifluoromethoxy)phenylhydrazone
FcεRI: high-affinity IgE receptor
Gab2: Grb2-associeted binder 1
HINT1: histidine triad nucleotide-binding protein 1
IgE: immunoglobulin E
IKK: IκB kinase
IL-6: Interlukin 6
IL-8: Interlukin 8
IP_3_: inositol trisphosphate
IP_3_R: inositol trisphosphate receptor
IκB: inhibitor of nuclear factor kappa B
JNK: c-Jun N-terminal kinases
LAD2: laboratory of allergic diseases 2
LAT: linker for activation of T cells
LysRS: lysyl-tRNA synthetase
M2PK: M2 pyruvate kinase
MAPK: mitogen-activated protein kinase
MCU: mitochondrial calcium uniporter
mDivi-1: mitochondrial division inhibitor 1
Mfn1: mitofusin1
Mfn2: mitofusin2
MITF: microphthalmia associated transcription factor
MLCK: myosin light chain kinase
mPTP: mitochondrial permeability transition pore
NAD: nicotinamide adenine dinucleotide
NADH: NAD hydrogen
NEMO: NF-κB essential modulator
NFAT: nuclear factors of activated T cell
NF-kB: nuclear factor- kappaB
NOX4: NADH oxidase
NTAL: non-T cell activation linker
OPA1: dynamin-like 120 kDa
Orai1: calcium release-activated calcium channel protein 1
OXPHOS: oxidative phosphorylation
p66shc: proteins having Src homology 2
PDC: pyruvate dehydrogenase complex
PDH: pyruvate dehydrogenase
PDH E1α: pyruvate dehydrogenase E1α subunit
PGC-1 α: peroxisome proliferator–activated receptor γ coactivator 1 α
PINK1: phosphatase and tensin homolog induced kinase 1
PIP_2_: phosphatidylinositol 4,5-bisphosphate
PKC: protein kinase C
PLCγ: phospholipase Cγ
RBL-2H3: rat basophilic leukemia
ROS: reactive oxygen species
SERCA: sarco/endoplasmic reticulum Ca^2+^-ATPase
siRNA: small interfering ribonucleic acid (RNA)
SNARE: soluble NSF attachment protein receptor
STAT3: signal transducer and activator of transcription 3
STIM1: stromal interaction molecule 1
Syk: spleen tyrosine kinase
TFEB: transcription factor EB
Th2: T-helper type 2
TNFα: tumor necrosis factor alpha
TRPC: Ca^2+^ transporters transient receptor potential canonical
TRPML1: mucolipin 1 TRP or MCOLN1 channel
UCP2: uncoupling protein 2
Uqcrfs1: ubiquinol-cytochrome C reductase, Rieske iron-sulfur polypeptide 1
VDAC: voltage-dependent anion channels.
