# Corrigendum: The Role Played by Mitochondria in FcϵRI-Dependent Mast Cell Activation

**DOI:** 10.3389/fimmu.2020.620293

**Published:** 2020-12-11

**Authors:** Maria A. Chelombitko, Boris V. Chernyak, Artem V. Fedorov, Roman A. Zinovkin, Ehud Razin, Lakhsmi Bhargavi Paruchuru

**Affiliations:** ^1^ Belozersky Institute of Physico-Chemical Biology, Lomonosov Moscow State University, Moscow, Russia; ^2^ Department of Cell Biology and Histology, Biology Faculty, Lomonosov Moscow State University, Moscow, Russia; ^3^ Institute of Molecular Medicine, I.M. Sechenov First Moscow State Medical University, Moscow, Russia; ^4^ Department of Biochemistry and Molecular Biology, School of Medicine, Hebrew University of Jerusalem, Jerusalem, Israel

**Keywords:** mast cell, mitochondria, FcϵRI-dependent activation, IgE, allergy

In the original article, there was an error. The statement that mitochondrial ROS inhibit the activity of NEMO is wrong. Mitochondrial ROS are crucial for the activation of the IKK-NEMO complex.

A correction has been made to the section** The Role Played by Mitochondria in the FcϵRI-Dependent Mast Cell Activation,** subsection **Mitochondrial ROS**, paragraph 6. The correct paragraph appears below.

Mitochondrial ROS can stimulate NF-κB signaling by activating the kinase (IKK) of the inhibitor of NF-κB (IκB), which promotes its proteasome degradation and induces nuclear translocation of NF-κB (81, 84). Mitochondrial ROS-dependent activation of IKK can be mediated by several mechanisms, including the formation of intermolecular disulfide bonds in NF-κB essential modulator (NEMO), a component of the IKK complex (85).

The authors apologize for this error and state that this does not change the scientific conclusions of the article in any way. The original article has been updated.
